# Supplementary material for: Identification and functional analysis of non-coding regulatory small RNA FenSr3 in Bacillus amyloliquefaciens LPB-18
Source: PeerJ. 2023 May 15;11:e15236. doi: 10.7717/peerj.15236 (PMC10194069; doi:10.7717/peerj.15236)
Supplement: Supplemental Information 4 [file peerj-11-15236-s004.zip › KO/CK-vs-T1_map/map04930.html]

KEGG PATHWAY: Type II diabetes mellitus - Reference pathway


|  |  |
| --- | --- |
| **Type II diabetes mellitus - Reference pathway** |  |

[
Pathway menu
| Organism menu
| Pathway entry
| Show description
| User data mapping
]

Insulin resistance is strongly associated with type II diabetes. "Diabetogenic" factors including FFA, TNFalpha and cellular stress induce insulin resistance through inhibition of IRS1 functions. Serine/threonine phosphorylation, interaction with SOCS, regulation of the expression, modification of the cellular localization, and degradation represent the molecular mechanisms stimulated by them. Various kinases (ERK, JNK, IKKbeta, PKCzeta, PKCtheta and mTOR) are involved in this process.
The development of type II diabetes requires impaired beta-cell function. Chronic hyperglycemia has been shown to induce multiple defects in beta-cells. Hyperglycemia has been proposed to lead to large amounts of reactive oxygen species (ROS) in beta-cells, with subsequent damage to cellular components including PDX-1. Loss of PDX-1, a critical regulator of insulin promoter activity, has also been proposed as an important mechanism leading to beta-cell dysfunction.
Although there is little doubt as to the importance of genetic factors in type II diabetes, genetic analysis is difficult due to complex interaction among multiple susceptibility genes and between genetic and environmental factors. Genetic studies have therefore given very diverse results. Kir6.2 and IRS are two of the candidate genes. It is known that Kir6.2 and IRS play central roles in insulin secretion and insulin signal transmission, respectively.

|  |  |  |
| --- | --- | --- |
| Reference pathway | 184% 150% 122% 100% 82% 67% 55% | 图片下载 |
